# Formulation of Multiple Diffraction by Trees and Buildings for Radio Propagation Predictions for Local Multipoint Distribution Service

**DOI:** 10.6028/jres.104.036

**Published:** 1999-12-01

**Authors:** Wei Zhang

**Affiliations:** National Institute of Standards and Technology, Gaithersburg, MD 20899-0001

**Keywords:** building, local multipoint distribution service (LMDS), microwave propagation, multiple diffraction, tree, vegetation, wireless communications

## Abstract

This paper presents a closed-form expression for multiple forward diffraction by rows of tree canopies and buildings applicable to the propagation predictions at centimeter and millimeter wavelengths for local multipoint distribution service (LMDS). The expression is derived from the uniform geometrical theory of diffraction and physical optics, as well as from some existing models for vegetation and buildings. When the transmitter antennas are sufficiently high, the attenuation of the buildings varies around the value of free space and the building effect is negligible, because a line-of-sight (LOS) propagation path between transmitter and over building-rooftop receiver antennas exists and plays a major role. The tree canopies which extend above the building rooftop heights block the LOS propagation path and cause additional signal attenuation. An existing study of the LMDS radio channel based on measurements came to the same conclusion. The attenuation effect of the buildings is significant if the transmitter antennas are not high enough.

## 1. Introduction

Since microwave and millimeter wave communications systems are considered for use in a local multipoint distribution service (LMDS) [1–3], it is necessary and important to develop propagation prediction tools to facilitate good system design leading to excellent service. It is appropriate to implement a high transmitter antenna and building rooftop receiver antennas for small cells, which provide line-of-sight (LOS) propagation paths between transmitter and receivers. As reported in a study of the LMDS radio channel [1], radio propagation impairments for LMDS systems should be studied. The effects of vegetation, buildings, and rain and other precipitation on radio propagation must be considered and included in the design of the system. Several models for attenuation in vegetation media are available [4]. The effects of rain and other precipitation on radio propagation have been studied for many years for satellite and terrestrial telecommunications and for radar remote sensing. However, the suitability of the existing models and results for LMDS systems needs to be investigated. In particular, tree canopies that extend above the building rooftop heights block LOS propagation paths and cause signal attenuation, depolarization, and multipath. For a wavelength λ much larger than the size of tree leaves and branches, e.g., 900 MHz, a theoretical model has been proposed to compute the diffraction effects of tree canopies and buildings [5]. Unfortunately, λ at centimeter and millimeter frequencies (1 cm at 30 GHz) may be on the order of sizes of tree leaves and branches and even much smaller. Therefore, the model proposed for use in mobile radio systems is not valid for LMDS propagation predictions [5]. There is a lack of engineering solutions for LMDS systems, and a need for better understanding of the propagation mechanism, especially for propagation environments including vegetation and buildings.

**Fig. 1:**
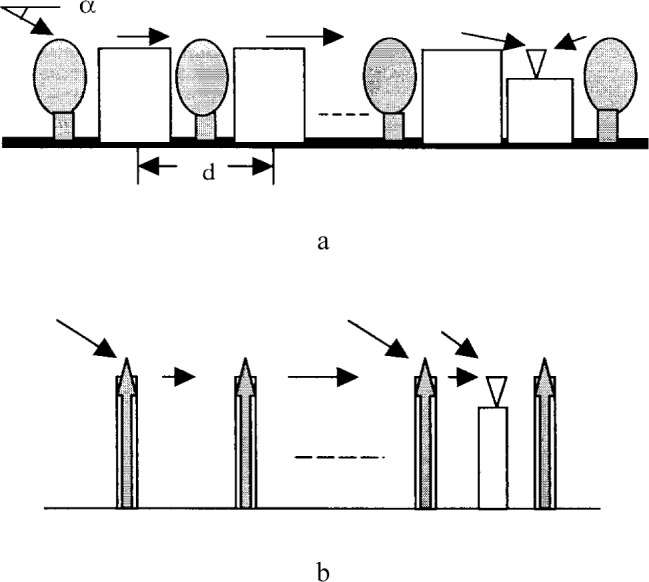
Radio propagation in the presence of rows of trees and buildings. (a) Diffraction by buildings and tree canopies extending above the building rooftop heights. (b) Diffraction modeling of Fig. 1 (a): a multiplication of a knife-edge diffraction and a tree attenuation and phase factor accounting for a row of buildings and a tree canopy above the building rooftop height.

This paper studies the diffraction of tree canopies that extend above the building rooftop heights and of buildings and explains the effects of diffraction on radio propagation. A closed-form expression for multiple diffraction by trees and buildings is presented in Sec. 2, derived from the uniform geometrical theory of diffraction (UTD) [6] and from physical optics (PO), as well as from existing models for vegetation [4,7]. It is known that UTD itself may become incorrect when the rooftop of each multiple diffraction building (modeled as an edge) lies in the transition zone of rooftops of the preceding diffraction buildings as indicated in Fig. 1. Because of this, PO-based results are applied for multiple edge diffraction near and in the transition zone [8,9]. The physical optics approximation is accurate in and near the transition zone but generally involves multiple dimension integration due to the multiple diffraction. The behavior of the closed-form diffraction formulation in and near the transition zone is examined and presented in Sec. 3. Numerical results of the formulation are presented and discussed in Sec. 4, along with their relevance to the LMDS radio channel. The entire work is summarized in Sec. 5.

## 2. Multiple Diffraction Formulation

This section presents the multiple forward diffraction expression for trees and buildings as represented in Fig. 1. The diffraction modeling of Fig. 1 (a) is indicated in Fig. 1 (b). A multiplication of a knife-edge diffraction and a tree attenuation and phase factor is used to account for the diffraction of a row of buildings and a tree canopy above the building rooftop height. The rows of buildings or trees are numbered 1 to *n*. There is no direct ray from transmitter to receiver, because the tree canopies extend above the average building rooftop height and block the LOS propagation path. The strongest ray suffers attenuation by the tree canopy.

Consider an incident plane wave *E*_0_ and the corresponding total field *E*_*n*+1_ at the reference point *n*+1 (receiver) as defined for the formulation of multiple diffraction in previous investigations [10,5]. Let *k*_0_ be the wave-number in free space, *α* be the elevation angle, *d* be the average separation distance between rows of trees or buildings, and $g=\sin\alpha \sqrt{d/\lambda }$ be a group parameter. In the range of *g* > 0, the closed-form expression for the field ratio *E*_*n*+1_/*E*_0_ or |*E*_*n*+1_/*E*_0_| is

$$\begin{array}{c} \frac{E_{n+1}}{E_{0}}=A\left(1+\frac{D_{s,h}}{\sqrt{d}}e^{-jk_{0}d(1-cos\alpha )}\frac{1-{\left(Ae^{-j\Delta k\Delta d}D_{s,h}^{c}e^{-jk_{0}d(1-cos\alpha )}/\sqrt{d}\right)}^{n}}{1-{\left(Ae^{-j\Delta k\Delta d}D_{s,h}^{c}e^{-jk_{0}d(1-cos\alpha )}/\sqrt{d}\right)}^{n}}\right)\\ A=e^{-L/8.686}\\ \Delta k=k_{0}(n_{R}-1) \end{array}$$ (1)

(using the results from Ref. [10] and including the effects of propagation through the trees where *A* and e^jΔ*k*Δ*d*^ are the attenuation and the phase factor of a tree in the canopy, respectively, Δ*d* is the average propagation path length (depth) through a tree in the canopy, *L* denotes the attenuation in dB due to vegetation (in excess of that of free space) available in the range of 10 GHz to 40 GHz [4], and *n*_R_ is the real part of the refractive index of leaves [7]. For the in-leaf and out-of-leaf states, Lis expressed as *L* = 0.39 *f*^0.39^ Δ*d*^0.25^ and *L* = 0.37*f*^0.18^ Δ*d*^0.59^, respectively, where *f* is the frequency in MHz and Δ*d* is in meters. From the results of Refs. [7] and [4], $n_{R}=\sqrt{\varepsilon^{\prime }+n_{I}^{2}}$, where *n*_I_ is the imaginary part of the refractive index derived from *n*_I_ = *L*/8.686 at Δ*d* = 1 *m* and ε′ is the real part of the relative permittivity written as ε′ = *A*′ − B′*m*_d_ with 0.1 ≤ *m*_d_ ≤ 0.5 [7]. Coefficients *A*′ and B′ are available in [7].

In the absence of a tree canopy, i.e., *A* = 1, Eq. (1) here becomes formula (4) of Ref. [11] for multiple building forward diffraction. As with the procedure presented in Refs. [11] and [12], the derivation of Eq. (1) here is similar to the derivation of formula (4) of Ref. [11]. The differences are: (1) the multiplication of each UTD building diffraction coefficient by the factor A of a tree canopy with an appropriate phase factor e^−jΔ*k*Δdcosα^ or e^−jΔ*k*Δd^, instead of a UTD diffraction coefficient alone; and (2) taking the strongest ray to be of the form $Ae^{-j\Delta k\Delta d\cos\alpha }\times e^{-jk_{0}nd\cos\alpha }$, instead of the direct ray of the form $e^{-jk_{0}nd\cos\alpha }$. Item (1) accounts for the diffraction by a row of trees and buildings. Item (2) is introduced for the absence of an LOS propagation path.

The UTD diffraction coefficients *D*_s,h_ and $D_{s,h}^{c}$in Eq. (1) are written as [6]

$$D_{s,h}\approx \frac{-e^{-j\pi /4}}{2\sqrt{2\pi k_{0}}}\left[\frac{F(X)}{\sin(\alpha /2)}\mp \frac{1}{-\cos(\alpha /2)}\right]$$ (2)

$$F(X)=\sqrt{\pi X}e^{j\pi /4+jX}-2j\sqrt{X}e^{jX}\int_{0}^{\sqrt{X}}e^{-j\tau^{2}}d\tau$$ (3)

$$\sqrt{X}=\sqrt{2k_{0}d}|\text{sin}(\alpha /2)|$$ (4)

$$D_{s,h}=\frac{-e^{-j\pi /4}}{2\sqrt{2\pi k_{0}}}\left[-\sqrt{\pi k_{0}d}\cdot e^{j\pi /4}\mp (-1)\right],$$ (5)

where the subscripts “s” and “h” of *D*^s,h^ and $D_{s,h}^{c}$denote soft and hard boundaries, respectively, and they take signs “−” and “+” on the right-hand side of Eqs. (2) and (5). The hard boundary corresponds to vertical polarization transmission and reception in the vertical plane. The transition function *F*(*X*) can be approximated by $F(X)\approx \sqrt{\pi X}e^{j\pi /4+jX}$ for *X* < 10^−3^ and *F*(*X*) ≈ 1 for *X* > 10. Many LMDS propagation environments would in fact have *X* > 10. For example, at 30 GHz, *d* = 40 *m*, and *α* ≥ 1.62°, Eq. (4) results in *X* > 10. Equation (1) may fail when *g* is small, especially as *g* → 0, corresponding to the grazing aspects of incidence and observations. The failure of UTD multiple diffraction may occur for *g* < 0.1 as stated in Ref. [12].

For $0\leq g\approx \alpha \sqrt{d/\lambda }<0.1$ and a grazing incidence of *α* → 0, the expression for *E*_*n*+1_/*E*_0_ is

$$\frac{E_{n+1}}{E_{0}}=A\left(1+\frac{D_{s,h}}{\sqrt{d}}e^{-jk_{0}d(1-cos\alpha )}\frac{1-A^{\gamma_{n}}/\sqrt{3n+1}}{{-D_{s,h}|}_{\alpha =0}/\sqrt{d}}\right)$$ (6)

$${D_{s,h}|}_{\alpha =0}=\frac{-e^{-j\pi /4}}{2\sqrt{2\pi k_{0}}}[\sqrt{2\pi k_{0}d}\cdot e^{j\pi /4}\mp (-1)],$$ (7)

where *γ*_*n*_ is a function of the number of rows of buildings *n*. The expression $1/\sqrt{3n+1}$ by Walfisch and Bertoni [8] was used here; it approximates $\Gamma (n+1/2)/(\sqrt{\pi }n!)$ derived in Ref. [9] which is valid for both hard and soft boundaries. The factor $1/\sqrt{3n+1}$ accounts for the multiple forward diffraction by *n* rows of buildings at grazing incidence, i.e., *E*_*n*+1_/*E*_0_ at *α* → 0. Equation (6) is derived by writing

$$\frac{E_{n+1}}{E_{0}}=A\left(1+\frac{D_{s,h}}{\sqrt{d}}e^{-jk_{0}d(1-\cos\alpha )}ℋ\right)$$ (8)

and determining ℋ when 0 ≤ *g* < 0.1. The hybrid function ℋ (including tree attenuation effects) comes from UTD and physical optics that is accurate in the transition zone. It takes the advantages of both methods. One may approximate Eq. (8) as

$$\frac{E_{n+1}}{E_{0}}=\frac{A^{1+\gamma_{n}}}{\sqrt{3n+1}}$$ (9)

for *g* → 0, i.e., *α* → 0 and *k*_0_ *d* (1 − cosα) → 0. Equation (9) is a multiplication of the multiple-building diffraction factor $1/\sqrt{3n+1}$ and the tree attenuation factor $A^{1+\gamma_{n}}$. The building factor $1/\sqrt{3n+1}$ is valid when the building separation distance is much larger than the wavelength (*d* ≫ λ); the tree attenuation factor *A* is valid at 10 GHz to 40 GHz [4]. Therefore, Eq. (9), which is an approximation, can hold for the range 20 GHz to 40 GHz. This means that ℋ can be approximated as

$$ℋ\approx \frac{1-A^{\gamma n}/\sqrt{3n+1}}{{-D_{s,h}|}_{\alpha =0}/\sqrt{d}}.$$ (10)

The function *γ*_*n*_ ranges from 0 to *n*−1, resulting in variations of tree attenuation. In the numerical calculations for this work, *γ*_*n*_ ≈ 0 was taken. This should be adequate to indicate the tree effects for LMDS systems that are suppose to use high transmitter antennas, providing LOS propagation paths in the absence of trees. In the presence of trees, LOS propagation conditions no longer exist. The strongest signal component that may exist is the one that propagates through only one tree, among a number of field components of multiple diffraction by trees and buildings that result in the total received signal. The elevation angle for high transmitter antennas is in the range *g* ≥ 0.1, where Eq. (1) applies. Equation (6) is valid for the range 0 ≤ *g* < 0.1. For small angle *α* and for a frequency of 900 MHz, i.e., gless than 0.06, the results of propagation loss due to trees and buildings were presented in Ref. [5]. It was seen there that the differences between propagation loss for trees and buildings and the loss in the absence of trees are nearly constant when the number of rows of trees or buildings is larger than about ten, 4 dB to 5 dB for wider trees. This corresponds to a constant value of *γ*_*n*_ for *n* ≥ 10.

Since *k*_0_ *d* ≫ 1 and *α* is small leading to cos(*α* / 2) ≈ 1, the second term on the right-hand sides of Eqs. (2), (5), and (7) is negligibly small compared with the first term. Therefore, both Eqs. (6) and (1) are dependent on but insensitive to the polarization (type of boundaries). Since *d* is in the range of about 30 *m* to 100 *m* or even larger, the condition *k*_0_ *d* ≫ 1 is valid at the microwave and millimeter wave frequencies being used for LMDS systems.

## 3. Asymptotic Expressions

To examine Eqs. (6) and (1) for small *α* ≈ 2 sin (*α* / 2) and small $g\approx \alpha \sqrt{d/\lambda }$, one may approximate Eq. (3) by

$$F(X)\approx [\sqrt{\pi X}-2X\;e^{j\pi /4}]\;e^{j\pi /4+jX},$$ (11)

where *X* ≈ π*g*^2^ is small [6]. For the purposes of practical engineering applications, the approximation of Eq. (11) can be taken for 0 ≤ *X* < 0.3 corresponding to 0 ≤ *g* < 0.3. One thus derives from Eq. (2)

$$D_{s,h}/\sqrt{d}\approx -1/2+ge^{j\pi /4}.$$ (12)

As a result, Eq. (1) can be approximated by

$$E_{n+1}/E_{0}\approx A\left(\frac{\sqrt{2}-Ae^{-j\Delta k\Delta d}}{2\sqrt{2}-Ae^{-j\Delta k\Delta d}}+\frac{2\sqrt{2}}{2\sqrt{2}-Ae^{-j\Delta k\Delta d}}ge^{j\pi /4}\right)$$ (13)

for 0.3 > *g* ≥ 0.1 and *n* sufficiently large (*n* ≥ 6 for *A* = 1 from numerical calculation). Further, Eq. (13) becomes

$$E_{n+1}/E_{0}\approx \frac{2\sqrt{2}}{2\sqrt{2}-Ae^{-j\Delta k\Delta d}}Ag$$ (14)

for $0.3>g>|1/2-2Ae^{-j\Delta k\Delta d}/\sqrt{2}|\geq 0.1$. Similarly, Eq. (6) can be approximated by

$$E_{n+1}/E_{0}\approx A\left(A^{\gamma_{n}}/\sqrt{3n+1}+2g(1-A^{\gamma_{n}}/\sqrt{3n+1})e^{j\pi /4}\right)$$ (15)

and becomes

$$E_{n+1}/E_{0}\approx 2Ag$$ (16)

for *n* ≫ 1. The settled field Q from Eq. (14) of Walfisch and Bertoni is

$$Q\approx 0.1{\left[\frac{\alpha \sqrt{d/\lambda }}{0.03}\right]}^{0.9}\approx 2.35g^{0.9}$$ (17)

for $\alpha \sqrt{d/\lambda }$ ranging about 0.02 to 0.4 [8]. If we consider the case *A* e^−jΔ*k*Δd^ = 1, i.e., the absence of trees, Eq. (14) (valid for 0.3 > *g* > 0.147) and Eq. (16) become

$$E_{n+1}/E_{0}\approx 1.55g$$ (18a)

$$E_{n+1}/E_{0}\approx 2g.$$ (18b)

It is interesting to see that these two asymptotic expressions are comparable with Eq. (17). For small *g*, the deviation of Eq. (18a) from Eq. (17) becomes large.

For *n* = 6 and *A* = 1, Eq. (15) becomes

$$E_{n+1}/E_{0}\approx 0.229+1.54ge^{j\pi /4}.$$ (19)

One can also write Eq. (13) as

$$E_{n+1}/E_{0}\approx 0.227+1.55ge^{j\pi /4}.$$ (20)

Incidentally, Eq. (1) smoothly approaches Eq. (6) when *g* → to 0.1 and *A* e^−jΔ*k*Δd^ = 1 for *n* ≤ 6. Both Eq. (6) and Eq. (1) also apply to a soft boundary that corresponds to horizontal polarization transmission and reception in the vertical plane.

## 4. Numerical Results

**Fig. 2:**
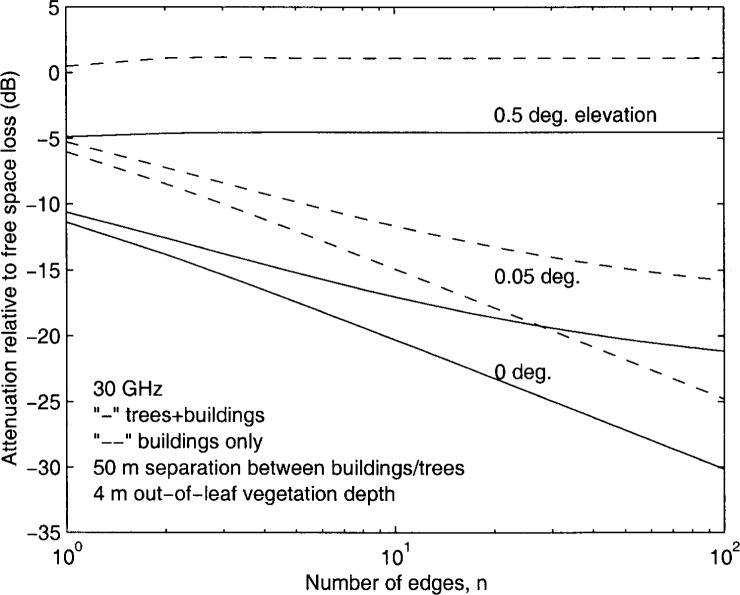
Attenuation relative to free space attenuation for a receiver at the rooftop of building number *n*+1 for a 50 *m* distance separation between trees and buildings.

**Fig. 3:**
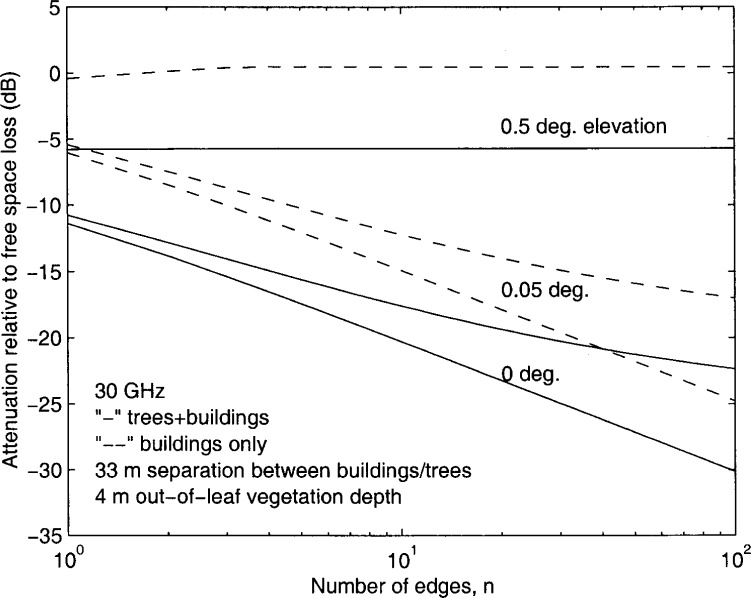
Attenuation relative to free space attenuation for the receiver at the rooftop of building number *n*+1 for a 33 *m* distance separation between trees and buildings.

Figures 2 and 3 present numerical results of Eqs. (1) and (6). The relative attenuation *A*_md_ in dB is derived from

$$A_{\text{md}}=20\log_{10}|E_{n+1}/E_{0}|.$$ (21)

Due to the presence of trees, the relative attenuation for trees and buildings is severe at *α* = 0.5°, where *g* takes the values 0.617 and 0.501 for *d* = 50 *m* and *d* = 33 *m*, respectively. In the absence of trees, the attenuation of buildings varies around the value of free space and the building effect is negligible, since an LOS propagation path between transmitter and receiver antennas exists and plays an important role for *g* > 0.4, corresponding to sufficiently high transmitter antennas [8,10]. The parameter *g* depends on frequency, elevation angle, and separation distance between buildings. The existence of an LOS path (a direct wave component) depends only on the elevation, i.e., for *α* > 0. The LOS propagation path becomes dominant when the elevation angle *α* is sufficiently large resulting in *g* > 0.4. In the presence of trees, the tree canopies that extend above the building rooftop heights block the LOS propagation path and cause additional signal attenuation. Based on the analysis of experimental data, a recent study of the LMDS radio channel concludes that a serious propagation impairment is signal attenuation caused by tree canopies [1].

It is seen that the differences between the relative attenuation for trees and buildings and the attenuation for the buildings only are insensitive to the number of edges modeling the trees and buildings. At *g* = 0, i.e., *α* = 0, Eq. (6) becomes $A^{1+\gamma_{n}}/\sqrt{3n+1}$, which is a multiplication of the diffraction factors for tree canopy and buildings. At an angle of *α* > 0, the absolute value of relative attenuation *A*_md_ increases as the separation distance *d* between trees or buildings decreases. Equation (6) generates the results for *α* = 0.05°, where *g* takes the values 0.0617 and 0.0501 for *d* = 50 *m* and *d* = 33 *m*, respectively. For a fixed elevation angle *α*, *g* decreases with *d*. It is known that the multiple building forward diffraction loss increases as the group parameter *g* decreases [8,10].

Since the depth Δ*d* is an input parameter, more numerical calculations for other values of Δ*d* are available. Also, there are several models of attenuation due to vegetation media at centimeter and millimeter wavelengths and these models can be taken as inputs of the present formulation of Eqs. (1) and (6) [4].

## 5. Conclusion

A closed-form expression for multiple forward diffraction by tree canopies and buildings has been derived and presented in order to make propagation predictions at centimeter and millimeter wavelengths for local multipoint distribution service. When the transmitter antennas are sufficiently high, the attenuation of the buildings varies around the value of free space and the building effect is negligible, because a line-of-sight propagation path between transmitter and over building rooftop receiver antennas exists and plays a major role. When trees extend above the building rooftop heights, they block the LOS propagation path and cause additional signal attenuation. The attenuation effect of the buildings is significant if the transmitter antennas are not high enough as *α* → 0. The attenuation due to rows of tree canopies and buildings increases as the separation distance between trees or buildings decreases. When enough measurement data become available, a comparison of the formulation with measurements and experimental studies of *γ*_*n*_ would lead to refinement or significant improvement of the prototype formulation given here.
